# Immunopathogenesis of neuropsychiatric systemic lupus erythematosus: current insights into inflammatory and thrombotic pathways

**DOI:** 10.1186/s12974-026-03920-1

**Published:** 2026-06-23

**Authors:** Igor Adamski, Anna Wardowska

**Affiliations:** Department of Rheumatology, Clinical Immunology, Geriatrics and Internal Medicine, Faculty of Medicine, Medical University of Gdańsk, Gdańsk, Poland

**Keywords:** SLE, NPSLE, CNS-NPSLE, PNS-NPSLE, Immunopathology, Blood-brain barrier

## Abstract

Systemic lupus erythematosus (SLE) is an autoimmune disease characterized by loss of immune tolerance, systemic inflammation, autoantibody production, and immune complex formation. Neuropsychiatric systemic lupus erythematosus (NPSLE) represents one of the most severe manifestations of the disease and may involve both the central and peripheral nervous systems. Its pathogenesis remains incompletely understood and reflects the interplay among systemic immune dysregulation, vascular injury, blood-brain and blood-CSF barrier dysfunction, and local neuroinflammatory processes.

Current evidence supports two partially overlapping pathogenic pathways: an autoimmune-inflammatory and a thrombo-ischemic pathway. These mechanisms promote disruption of the blood-brain barrier, infiltration of immune cells into the central nervous system, activation of microglia, and neuronal dysfunction. Increasing evidence also highlights the role of neutrophil extracellular traps, complement activation, type I interferon signaling, and autoreactive lymphocytes in driving neuroinflammation.

The heterogeneity of clinical manifestations and the diversity of underlying mechanisms make NPSLE a major diagnostic and therapeutic challenge. This review summarizes current knowledge on the immunopathogenesis of NPSLE, with particular attention to the strength of evidence supporting individual mechanisms. Human clinical studies provide important associative data, whereas animal models and cell-based systems offer mechanistic insight into selected pathways. We also discuss emerging biomarkers, limitations of current evidence, and potential therapeutic targets that may improve future diagnosis, stratification, and management of NPSLE.

## Introduction

 Systemic lupus erythematosus (SLE) is a chronic autoimmune disease characterized by a loss of immunological tolerance to self-antigens, leading to the production of autoantibodies and the formation of immune complexes. Deposition of these complexes in tissues triggers complement activation and inflammatory responses, resulting in damage to multiple organs. The clinical presentation of SLE varies considerably due to a wide spectrum of organ involvement [1]. Neuropsychiatric systemic lupus erythematosus (NPSLE) represents one of the most severe manifestations of this disease. Depending on the diagnostic criteria used, study design, and attribution models applied to distinguish primary NPSLE from secondary neurological complications, neuropsychiatric manifestations have been reported in approximately 12% to 95% of patients with SLE [2]. The clinical diagnosis of NPSLE is often delayed, leading to an underestimation of its prevalence and suboptimal management of affected patients [2]. Neurological manifestations of SLE represent a serious clinical complication, which may result in deterioration of motor control, cognitive impairment, and reduced quality of life, and are associated with an increased risk of organ damage and mortality [3, 4].

Several factors increase the risk of developing NPSLE in patients with SLE. Chronic systemic inflammation is considered one of the factors that may facilitate involvement of additional organs, including the nervous system. However, in only about 50% of cases, the diagnosis of NPSLE usually correlates with increased activity of the underlying disease [5]. Previous neuropsychiatric events, such as ischemic stroke, epileptic seizure, or psychosis, may predispose patients to exacerbation of NPSLE symptoms. The presence of antiphospholipid antibodies (APLAs) and antiphospholipid syndrome (APS) is also associated with an increased risk of nervous system involvement, particularly cerebrovascular events [5–7]. Moreover, poor prognosis and increased mortality of NPSLE patients are associated with high SLE activity, young age, organ damage present at the time of neuropsychiatric symptoms onset, and coexisting APS [8].

Approximately 60% of patients with SLE develop neuropsychiatric symptoms within the first year after diagnosis [5]. Therefore, the diagnosis of SLE should be followed by careful evaluation of potential nervous system involvement [5, 7, 9]. In some cases, however, neuropsychiatric manifestations may represent the initial presentation of SLE, further complicating the diagnostic process and delaying appropriate treatment [4, 5, 9]. Consequently, early identification of risk factors and active screening for neuropsychiatric symptoms are essential for timely recognition of NPSLE [5, 7].

To summarize the current state of knowledge, we reviewed the available literature on the immunopathogenesis of NPSLE, with particular attention to inflammatory, thrombotic, vascular, barrier-related, and neuroimmune mechanisms. The literature was evaluated with regard to the type and strength of supporting evidence, including clinical cohort studies, biomarker analyses, cerebrospinal fluid (CSF) studies, neuroimaging, histopathological reports, animal models, and in vitro or ex vivo experimental systems. Human observational studies were interpreted primarily as evidence of association between immune, vascular, neurological, and psychiatric abnormalities, whereas animal models and cell-based experiments were considered to provide mechanistic support for selected pathogenic pathways.

Accordingly, throughout this review, causal interpretations are restricted to mechanisms supported by experimental or functional evidence, whereas findings derived primarily from human observational studies are presented as associations. This approach is intended to distinguish established clinical observations from biologically plausible mechanisms that still require validation in human NPSLE. Particular emphasis is placed on complement activation, disruption of the blood-brain barrier (BBB) and blood-CSF barrier, endothelial injury, NETosis, type I IFN signaling, lymphocyte recruitment, microglial activation, and autoantibody-mediated neuronal dysfunction.

## Literature search strategy

A structured literature search was performed to identify studies relevant to the immunopathogenesis, clinical manifestations, biomarkers, and experimental models of neuropsychiatric systemic lupus erythematosus (NPSLE). The search was conducted in PubMed/MEDLINE and supplemented by screening the reference lists of relevant original articles, reviews, systematic reviews, meta-analyses, and international recommendations. The search was limited to articles published in English and was last updated on 10 May 2026.

The search strategy combined terms related to SLE and neuropsychiatric involvement with terms describing immune, vascular, barrier-related, and neuroinflammatory mechanisms. The following keywords and their combinations were used: “systemic lupus erythematosus”, “SLE”, “neuropsychiatric systemic lupus erythematosus”, “NPSLE”, “central nervous system lupus”, “peripheral nervous system lupus”, “autoantibodies”, “antiphospholipid antibodies”, “complement”, “C5a”, “C5b-9”, “immune complexes”, “blood–brain barrier”, “blood–CSF barrier”, “choroid plexus”, “NETosis”, “low-density granulocytes”, “type I interferon”, “endothelial dysfunction”, “vasculopathy”, “vasculitis”, “thrombosis”, “microglia”, “astrocytes”, “neuroinflammation”, and “murine models”.

Original clinical studies, cohort studies, CSF biomarker analyses, neuroimaging studies, histopathological and autopsy reports, animal studies, in vitro and ex vivo mechanistic studies, systematic reviews, meta-analyses, narrative reviews, and expert recommendations were considered eligible if they addressed NPSLE pathogenesis, clinical attribution, immune mechanisms, vascular injury, nervous system involvement, biomarkers, or therapeutic implications. Studies focusing on systemic SLE without direct relevance to nervous system involvement were included only when they addressed mechanisms applicable to NPSLE, such as complement activation, NETosis, type I IFN signaling, endothelial dysfunction, APLAs-mediated thrombosis, or lymphocyte dysregulation.

The included literature was narratively synthesized. As outlined in the Introduction, particular attention was paid to the source and strength of evidence, distinguishing human observational associations from mechanisms supported by animal models or functional cell-based studies.

## Primary and secondary NPSLE

**Fig. 1:**
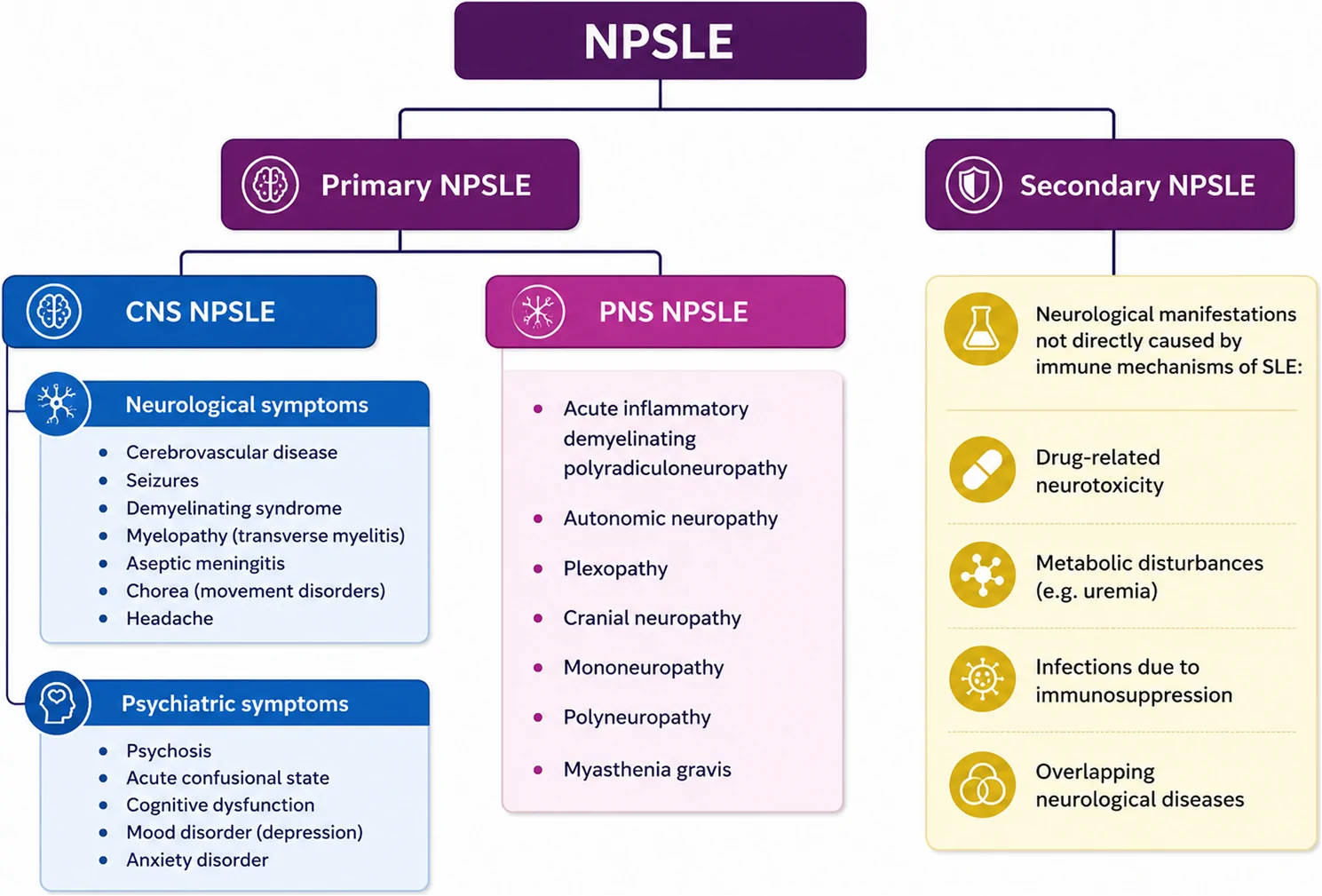
Classification of neuropsychiatric systemic lupus erythematosus (NPSLE) according to the American College of Rheumatology (ACR) nomenclature. NPSLE can be divided into primary and secondary forms. Primary NPSLE involves autoimmune-mediated central and peripheral nervous system manifestations, whereas secondary NPSLE refers to neurological manifestations caused by non-SLE-specific factors, such as treatment-related toxicity, metabolic disturbances, infections, or overlapping neurological diseases

Based on etiology and underlying mechanisms, NPSLE can be classified into primary and secondary forms (Fig. 1). Primary NPSLE refers to neuropsychiatric manifestations directly related to SLE, attributed to immune-mediated mechanisms, including autoimmune-inflammatory, thrombo-ischemic, vascular, and barrier-related processes. These mechanisms may involve both the central nervous system (CNS-NPSLE) and the peripheral nervous system (PNS-NPSLE). In this context, treatment usually relies on immunosuppressive, anti-inflammatory, anticoagulant, or antiplatelet strategies, depending on the presumed dominant pathogenic mechanism and clinical phenotype.

In contrast, secondary NPSLE refers to neurological or psychiatric manifestations resulting from causes other than direct immune-mediated nervous system involvement. These may include disease-related systemic complications, such as lupus nephritis-associated uremia, treatment-related adverse effects, metabolic disturbances, infections, or other comorbid conditions. Therefore, the therapeutic approach should be tailored to the specific cause of the neurological or psychiatric manifestations [10]. This review focuses primarily on primary NPSLE, with particular emphasis on autoimmune-inflammatory and thrombo-ischemic mechanisms.

Like SLE itself, NPSLE presents with marked clinical heterogeneity and may affect multiple functional components of both the central and the peripheral nervous system. This heterogeneity makes diagnosis and attribution particularly challenging. To address this issue, the American College of Rheumatology (ACR) proposed a standardized nomenclature system for NPSLE in 1999 [11]. Based on analyses of epidemiological and clinical data, this nomenclature lists 19 neuropsychiatric symptoms associated with SLE, including both CNS and PNS manifestations (Fig. 1). Importantly, the ACR classification is a nomenclature and case-definition system rather than a severity scale. Individual manifestations may occur in both mild and severe forms of NPSLE [2, 6, 11]. However, severe manifestations, such as cerebrovascular disease, acute confusional state, seizures, myelopathy, and psychosis, are generally associated with poorer prognosis and higher mortality [5, 7, 11]. Furthermore, clinical manifestations observed in patients with NPSLE are not strictly limited to those included in the ACR nomenclature. Nevertheless, manifestations outside this classification occur relatively infrequently in the overall NPSLE population [5, 7, 11].

## Pathomechanisms of NPSLE

### CNS-NPSLE

Central nervous system (CNS) involvement is generally considered the most clinically relevant component of NPSLE because it includes severe manifestations such as cerebrovascular disease, seizures, acute confusional state, psychosis, and cognitive dysfunction, whereas peripheral nervous system (PNS) involvement is less frequently reported and remains comparatively underexplored [5, 6, 8, 11–13]. The pathophysiological mechanisms underlying CNS-NPSLE can be broadly divided into two major pathogenic pathways: the autoimmune-inflammatory pathway and the thrombo-ischemic pathway. Differences in these mechanisms largely determine whether a given manifestation presents as focal or diffuse neuropsychiatric involvement [6, 8, 14]. The autoimmune-inflammatory pathway is more commonly associated with diffuse neuropsychiatric manifestations, whereas the thrombo-ischemic pathway is more frequently linked to focal CNS involvement. However, increasing evidence suggests substantial overlap between these mechanisms, and both pathways may contribute to focal as well as diffuse forms of NPSLE. Their interplay likely underlies the marked heterogeneity of clinical presentation observed in patients with NPSLE.

The autoimmune-inflammatory pathway is primarily linked to immune-mediated neuroinflammation involving autoantibodies directed against neuronal, glial, synaptic, endothelial, and systemic antigens, whereas the thrombo-ischemic pathway is associated with vascular injury, endothelial dysfunction, and prothrombotic processes. Thus, focal manifestations are more likely to arise when vascular occlusion, thrombosis, vasculopathy, or localized ischemic injury predominates, whereas diffuse manifestations are more likely to reflect widespread BBB or blood–CSF barrier dysfunction, cytokine-mediated neuroinflammation, autoantibody-mediated neuronal or glial dysfunction, and microglial activation. Nevertheless, these mechanisms frequently overlap, and a single clinical phenotype may result from both vascular and inflammatory processes.

Antibody-mediated mechanisms are discussed in more detail in the following section and summarized in Table 1.

**Table 1:** Autoantibodies associated with neuropsychiatric systemic lupus erythematosus (NPSLE)

Autoantibody	Target / Mechanism	Main Clinical Associations	Frequency	NPSLE phenotype	Ref.
Anti-NMDAR (anti-NMDA receptor)	Cross-reactivity with NR2A/NR2B NMDA receptor subunits → glutamate-mediated excitotoxicity	Mood disorders, anxiety, cognitive dysfunction	30–35%	Diffuse NPSLE, especially psychiatric manifestations	[2, 15–17]
Anti-MAP2	Targeting neuronal cytoskeletal protein MAP2 → disruption of neuronal structure and signaling	Psychosis, seizures, depression	17–33%	CNS-NPSLE	[2, 18–21]
Anti-ribosomal P (anti-RP)	Binding to neuronal ribosomal P proteins in limbic structures → neuronal dysfunction	Psychosis, seizures, mood disorders	46%	Diffuse NPSLE; associated with severe disease	[2, 15, 22, 23]
Anti-endothelial cell antibodies (AECA)	Endothelial activation and adhesion molecule expression → vascular inflammation and thrombosis	Psychosis, depression	60%	Vasculopathic / thrombotic NPSLE	[2, 15, 24]
Anti-ganglioside antibodies	Targeting neuronal membrane gangliosides → impaired neural signal transmission	Peripheral neuropathy, cognitive impairment	33%	PNS-NPSLE	[15, 25, 26]
Anti-aquaporin-4 (AQP4)	Astrocyte damage and BBB dysfunction	Demyelinating syndromes (NMOSD-like)	3% overall; up to 27% in demyelinating NPSLE	Focal demyelinating NPSLE	[2, 15, 27]

*NMDAR* N-methyl-D-aspartate receptor, *NR2A/NR2B* Subunit proteins of NMDAR, *MAP2* Microtubule-associated protein 2

#### Autoimmune-inflammatory pathway

The autoimmune-inflammatory pathway is considered particularly relevant to the diffuse form of NPSLE, although the strength of evidence varies across the proposed mechanisms. Clinical studies and systematic reviews have associated NPSLE with a broad spectrum of autoantibodies directed against both brain-associated antigens, including neuronal, ganglioside, synaptosomal, glial, and N-methyl-D-aspartate receptor antigens, and systemic antigens, including nuclear, cytoplasmic, phospholipid, and endothelial cell antigens [28, 29]. These autoantibodies may contribute to CNS-NPSLE through several partially overlapping mechanisms, including direct interference with neuronal function, glutamate-mediated excitotoxicity, endothelial activation, vascular inflammation, and immune-mediated disruption of CNS barriers. However, in human studies, these autoantibodies most often represent associative biomarkers, whereas animal models and cell-based studies provide more direct support for their potential pathogenic effects. The mechanistic diversity is reflected in the broad clinical spectrum of CNS involvement, ranging from mood disorders, psychosis, seizures, and cognitive dysfunction to focal demyelinating manifestations. The main antibodies implicated in the neuropsychiatric manifestations of SLE, together with their proposed targets, mechanisms of action, clinical associations, frequencies, and predominant neuropsychiatric phenotypes, are presented in Table 1.

Clinical evidence supports BBB dysfunction in at least a subset of patients with NPSLE. CSF studies have reported increased levels of inflammatory mediators, including IFN-α, IL-1β, IL-6, IL-8, and IFN-γ, as well as albumin, matrix metalloproteinase-9, and immunoglobulins (e.g., ANA, anti-NMDAR, anti-ribosomal P, and antiphospholipid antibodies) in patients with neuropsychiatric manifestations [21, 30–34]. These findings indicate altered barrier permeability, intrathecal immune activation, or both; however, they do not by themselves establish whether BBB disruption is a primary pathogenic event or a secondary consequence of ongoing CNS inflammation.

Low-density granulocyte (LDG)-mediated NETosis has been proposed as an upstream mechanism contributing to autoantigen exposure in SLE. NETs provide a source of nuclear and cytoplasmic autoantigens, including nucleic acid and centromere components, which may promote polyclonal B-cell activation and autoantibody production [35–41]. These processes facilitate the formation of circulating immune complexes, a well-established mechanism of systemic organ damage in SLE [40, 42–46]. In the context of NPSLE, the contribution of NETosis to CNS injury is biologically plausible and supported by experimental and translational data, but direct demonstration of this sequence in human CNS tissue remains limited.

When deposited in target tissues, including the CNS-associated barriers such as the choroid plexus, immune complexes may contribute to local immune activation. Mechanistic studies indicate that complement components can be recruited to immune complexes, particularly C1q, which binds to the Fc fragment of IgG and IgM and initiates the classical complement pathway [42–45, 47]. These findings provide mechanistic support for a link between immune complex deposition, complement activation, endothelial or epithelial activation, and BBB impairment. In human NPSLE, however, these mechanisms are mostly inferred from CSF biomarker studies, complement-related abnormalities, histopathological findings, and markers of endothelial activation rather than from direct longitudinal evidence.

Dendritic cells may further amplify the autoimmune-inflammatory cascade. In vitro studies have shown that lupus autoantibody-DNA immune complexes can activate dendritic cells through Fc receptor- and TLR9-dependent pathways, leading to type I IFN production [48]. Dendritic cell-derived IFN-α may then reinforce local and systemic immune activation. In parallel, inflammatory chemokines such as CCL2, linked to innate immune signaling pathways, including Nod-like receptor activation, may contribute to endothelial dysfunction and barrier damage. Experimental data suggest that CCL2-driven inflammatory signaling can promote pyroptosis in vascular endothelial cells, thereby further compromising BBB integrity [49]. These findings provide mechanistic support for the involvement of innate immunity activation in barrier disruption, although the precise contribution of dendritic cell-derived mediators, CCL2 signaling, and endothelial pyroptosis to human NPSLE remains to be fully established.

Deposited immune complexes and infiltrating immune cells can stimulate the secretion of proinflammatory cytokines, including IL-1, IL-6, IL-8, IFN-γ, IL-17, IL-23, and TGF-β [30, 32, 50–53]. Clinical CSF studies support the presence of elevated inflammatory mediators in NPSLE, whereas experimental studies suggest that cytokines such as IL-17 can directly promote BBB disruption and leukocyte migration across brain endothelial barriers [54–56]. Monocytes, neutrophils, and lymphocytes may migrate into the choroid plexus in response to chemotactic gradients, leading to the production of additional cytokines, thereby amplifying local inflammation. Thus, cytokine-mediated barrier dysfunction is supported by both clinical associations and experimental mechanistic evidence.

The cellular interactions involved in these processes are discussed in more detail in the following sections of this review.

#### Thrombo-ischemic pathway

The thrombo-ischemic pathway is primarily associated with vascular injury and focal forms of CNS-NPSLE, particularly those affecting small cerebral vessels. Clinical, histopathological, and imaging studies indicate that endothelial dysfunction, vasculitis, vasculopathy, immune complex deposition, and vascular wall thickening may contribute to cerebrovascular injury in SLE [8, 9, 57–60]. Compared with the autoimmune-inflammatory pathway, this mechanism is more directly linked to ischemic events, microvascular occlusion, and thrombotic complications, although inflammatory and thrombotic mechanisms frequently overlap at the level of the neurovascular unit.

Similarly to the autoimmune-inflammatory pathway, LDG-mediated NETosis may provide autoantigens, including nucleic acids and centromere components, that are recognized by circulating autoantibodies such as ANAs [35–37, 41]. The resulting immune complexes may be deposited within the CNS vasculature and contribute to endothelial activation and vascular injury. In human NPSLE, however, the sequence is usually inferred from systemic immune activation, vascular pathology, and biomarker studies rather than directly demonstrated within CNS tissue.

In addition to immune complex deposition, APS is among the best-established contributors to thrombotic and ischemic manifestations in SLE. APS is characterized by arterial or venous thrombosis associated with elevated levels of APLAs [61]. APS coexists with SLE in a substantial subset of patients, whereas population-based studies indicate that APS is rare in the general population, with an estimated prevalence of approximately 50 per 100,000 individuals [62–64]. Circulating APLAs, including lupus anticoagulant, anti-cardiolipin antibodies, and anti-β2-glycoprotein I antibodies, bind to phospholipid-associated antigens and endothelial cells within the vasculature, including CNS vessels [3, 8]. Clinical associations between APLAs and thrombotic events are well established, whereas experimental and mechanistic studies support their ability to activate endothelial cells, monocytes, and platelets, thereby promoting thrombosis, vascular inflammation, and endothelial injury [57, 61, 65, 66].

Complement activation may amplify these processes. Complement components, particularly C1q, can bind to immune complexes and enhance inflammatory and thrombotic responses [42–45, 47]. Endothelial cells acquire an activated phenotype characterized by increased expression of adhesion molecules such as VCAM-1, facilitating leukocyte adhesion and migration. Inflammatory mediators, including IL-1, IL-6, IL-8, and MMP-9, may further contribute to vascular damage and thrombus formation [57, 65–68]. Monocyte and leukocyte infiltration into vessel walls may promote vasculitis and further enhance thrombosis. These mechanisms may result in luminal narrowing, non-inflammatory small-vessel vasculopathy, microvascular occlusion, multiple microinfarctions, embolic events, and intracerebral microhemorrhages [8, 9, 58–60, 69]. Vasculitis can also contribute to BBB disruption, although its contribution to barrier failure is generally considered less prominent than that of the autoimmune-inflammatory pathway.

Importantly, APLAs have also been associated with diffuse neuropsychiatric manifestations, including seizures, chorea, and cognitive impairment [61, 70, 71]. These observations suggest that their pathogenic role may extend beyond purely prothrombotic effects. However, because much of this evidence is derived from clinical associations, direct causality in diffuse NPSLE remains difficult to establish. Anti-endothelial cell antibodies may also contribute to endothelial dysfunction, inflammatory cytokine production, and apoptosis. They have been associated with psychiatric manifestations in SLE and may induce the production of proinflammatory cytokines, including IL-1, IL-6, IL-8, IL-17, and TNF, thereby promoting endothelial activation and vascular injury [3, 24, 57, 65]. These findings are supported by both clinical observations and experimental studies of endothelial activation.

Furthermore, APLAs and anti-endothelial cell antibodies may contribute to accelerated atherosclerosis, which represents an independent risk factor for cerebral ischemia. Although several cardiovascular risk factors observed in patients with SLE, including dyslipidemia, smoking, and aging, overlap with those present in the general population, chronic systemic inflammation and persistent immune activation are thought to substantially accelerate vascular injury and atherosclerosis development [65, 66]. Elevated levels of proinflammatory cytokines and IFNs (type I and II) may activate innate immune responses associated with oxidative stress, adipokine dysregulation, dysfunctional lipid metabolism, and increased production of matrix metalloproteinases and elastase. These mechanisms can promote elastin degradation, release of fibroblast growth factor, endothelial dysfunction, apoptosis, extracellular matrix remodeling, and vascular wall damage [66, 72–77].

Adaptive immune mechanisms additionally contribute to vascular pathology through immune complex deposition. Circulating immune complexes composed of ANAs bound to DNA, RNA, and histones recruit complement component C1q, leading to complement activation and enhanced chemotactic recruitment of neutrophils and monocytes [42–46, 78]. These cells may subsequently release IL-1, TNF-α, IL-6, and reactive oxygen species, thereby amplifying local vascular inflammation and tissue injury. In parallel, complexes formed between oxidized low-density lipoprotein, APLAs, β2-glycoprotein I, and anti-β2-glycoprotein I antibodies may further accelerate atherosclerotic processes [65, 66]. These mechanisms have been associated with complement activation, as reflected by CH50 activity, and may contribute to cerebrovascular dysfunction in patients with SLE [66, 72–76].

Clinical epidemiological studies indicate that atherosclerosis develops earlier and more frequently in patients with SLE than in the general population [66]. Premenopausal women aged 35–44 years with SLE have been reported to exhibit up to a 50-fold higher risk of myocardial infarction compared with age-matched controls, supporting the concept of accelerated vascular aging and premature atherosclerosis development [79]. Moreover, atherosclerosis in SLE has been associated with a 2–5-fold increased risk of subsequent cardiac, cerebrovascular, and peripheral arterial ischemic events, as well as increased mortality [72]. Among individuals younger than 50 years of age, the risk of stroke is approximately eightfold higher in patients positive for antiphospholipid antibodies than in seronegative individuals [80]. Consequently, the risk of focal NPSLE manifestations is higher in patients with elevated APLAs than in seronegative individuals within the SLE population [70, 71]. These data provide strong clinical evidence for increased vascular risk in SLE and support an association between APLAs and cerebrovascular manifestations of NPSLE. However, the relative contribution of traditional cardiovascular risk factors, chronic inflammation, APLAs, complement activation, and endothelial dysfunction may vary among patients.

Importantly, despite their distinct pathogenic features, thrombotic and inflammatory mechanisms closely interact at the level of the neurovascular unit and BBB integrity. Therefore, focal and diffuse manifestations of CNS-NPSLE should not be viewed as completely separate entities, but rather as partially overlapping consequences of immune-mediated vascular, inflammatory, thrombotic, and barrier-related injury.

#### Blood-brain barrier disruption and neuroinflammation

The autoimmune-inflammatory and thrombo-ischemic pathways converge at the level of the BBB, whose disruption is considered a critical step in the development of CNS-NPSLE. Evidence supporting BBB dysfunction derives from several complementary sources, including CSF biomarker studies, neuroimaging, histopathological reports, animal models, and cell-based experimental systems. However, the level of evidence differs across these approaches, and direct causal relationships in human NPSLE remain difficult to establish.

Neurohistopathological studies have demonstrated features of both vasculopathy and inflammation, as well as thrombi [58]. Only a limited number of studies have investigated brain histopathology in patients with NPSLE, largely because CNS tissue samples are rarely available. Nevertheless, available reports most frequently describe vasculopathic alterations, including extensive microinfarctions, diffuse small-vessel injury, and perivascular lymphocytic infiltrates [59]. Case-based autopsy studies have also reported immune complex vasculitis, leukocyte accumulation, platelet deposition, and small-vessel occlusion in severe NPSLE [9, 60]. These findings provide relatively direct pathological evidence of vascular and inflammatory CNS injury, although they are limited by small sample size and overrepresentation of severe cases.

BBB disruption may occur as a downstream consequence of both autoimmune-inflammatory and thrombo-ischemic mechanisms. Endothelial dysfunction associated with vasculitis, vasculopathy, immune complex deposition, complement activation, and cytokine-mediated injury can increase BBB permeability, facilitating the entry of immune complexes, cytokines, autoantibodies, and immune cells into the CNS [31, 57, 81–84]. Clinical CSF studies support the presence of barrier dysfunction in at least a subset of patients with NPSLE, including evidence of distinct BBB disruption profiles when compared with other neuroinflammatory diseases; however, they cannot always distinguish whether BBB disruption precedes neuroinflammation or occurs secondary to established CNS injury [21, 31–33, 85].

The choroid plexus, which forms the blood–CSF barrier, represents an additional critical gateway for immune cell entry into the CNS. Histopathological studies have demonstrated immunoglobulin deposition and lymphocytic infiltration in the choroid plexus of patients with SLE [86]. Experimental studies further support the involvement of CNS barrier-associated structures by demonstrating leukocyte adhesion, inflammatory cell infiltration, and immune activation within the choroid plexus and adjacent vascular compartments [86–88]. Neuroimaging studies have reported increased choroid plexus volume, which correlates with disease activity measured by SLEDAI, white matter lesion burden, and APLAs positivity, supporting its potential role as a neuroinflammatory biomarker in NPSLE [86, 89, 90]. These imaging findings are clinically informative, although they should be interpreted as associations rather than direct evidence of causality.

The ongoing inflammatory response within the choroid plexus and cerebral vasculature may promote migration and proliferation of lymphocytes, potentially contributing to the formation of tertiary lymphoid-like structures and further destabilization of BBB integrity. Increased expression of endothelial adhesion molecules, including ICAM-1, VCAM-1, and PECAM-1, facilitates leukocyte adhesion and transendothelial migration across the BBB [57, 81–83]. Elevated circulating adhesion molecules and endothelial activation markers in patients with NPSLE further support the presence of systemic endothelial activation and possible BBB dysfunction, although direct demonstration of these processes within the human CNS remains limited.

BBB and blood–CSF barrier disruption enables infiltration of monocytes, neutrophils, and lymphocytes into the CNS, as well as diffusion of autoantibodies, cytokines, and immune complexes into the brain parenchyma and CSF. Increased barrier permeability may also facilitate exposure of neuronal antigens to circulating autoantibodies, thereby amplifying local autoimmune responses. This mechanism is biologically plausible and supported by experimental models, although its direct demonstration in human NPSLE remains challenging.

Within the CNS, infiltrating immune mediators and autoantibodies can stimulate activation of microglia and astrocytes, leading to the production of neurotoxic mediators such as IL-6, TNF-α, IL-1β, IL-18, and reactive oxygen species [86, 91–96]. These inflammatory mediators may contribute to neuronal dysfunction, demyelination, synaptic injury, excitotoxicity, and sustained neuroinflammation. Both focal and diffuse forms of CNS-NPSLE have been associated with elevated concentrations of proinflammatory cytokines in CSF [30, 32]. These CSF findings support an inflammatory component of CNS involvement, although cytokine elevations are not necessarily specific for individual neuropsychiatric manifestations.

Diffuse neuropsychiatric manifestations frequently include psychiatric symptoms such as depression, anxiety, psychosis, and cognitive impairment. These manifestations have been linked to pathological processes involving the hippocampus, a brain region characterized by continuous neurogenesis and high neuroplasticity [92, 93, 97]. However, the relationship between hippocampal dysfunction and clinical symptoms in NPSLE is complex and likely reflects the combined effects of inflammation, vascular injury, autoantibody-mediated mechanisms, impaired BBB integrity, and altered neural network activity.

Experimental studies provide more direct mechanistic support for the role of neuroinflammation and microglial activation in NPSLE. Lupus-prone mouse models, particularly MRL/lpr mice, have demonstrated early behavioral abnormalities, anxiety-like behavior, cognitive dysfunction, and depressive-like phenotypes that may precede the development of severe NPSLE manifestations [97]. More specifically, in MRL/lpr lupus-prone mice, increased CD40 expression on hippocampal microglia was associated with cognitive dysfunction, supporting a mechanistic link between microglial activation and NPSLE-like neurocognitive impairment [93]. These observations suggest that CNS involvement can occur independently of advanced peripheral organ damage and support the concept of early neuroimmune dysregulation in NPSLE. In murine models, hippocampal microglial activation has been linked to synaptic alterations, neuronal stripping, and broader NPSLE-like manifestations [93, 94, 98, 99].

Type I IFN signaling has also been implicated in microglial activation and neuroinflammation. Experimental and translational studies indicate that peripheral type I IFN responses can alter microglial activation states and may contribute to synaptic and neuronal dysfunction in SLE-related neuroinflammation [94, 98–100]. These data support a plausible pathway linking systemic IFN activation with CNS dysfunction, although further validation in human NPSLE is required. In patients with NPSLE, functional MRI studies have demonstrated increased activation of compensatory neural pathways during memory tasks, suggesting dysfunction of primary neural circuits involved in cognition [27]. While these clinical imaging data do not prove microglial causality, they are consistent with experimental findings implicating hippocampal neuroinflammation in cognitive dysfunction.

Circulating immune complexes and IgG derived from patients with SLE have been shown to activate microglia through Fc receptor-mediated signaling pathways in experimental systems, thereby amplifying CNS inflammation [95, 96]. Intracerebroventricular administration of SLE serum in mice has also been reported to induce microglial activation and leukocyte adhesion in the cerebral microvasculature [86]. These findings suggest that SLE activity may influence CNS inflammation through antibody-mediated microglial activation, particularly under conditions of impaired barrier integrity. Pharmacological or pathway-directed interventions in lupus-prone mice provide additional mechanistic support for the pathogenic role of microglia and inflammatory signaling in NPSLE-like manifestations [94, 98, 99]. However, translation of these findings to human disease requires caution, because animal models reproduce selected aspects of NPSLE rather than the full clinical, immunological, and vascular complexity of the human condition.

Taken together, current evidence supports a model in which BBB and blood–CSF barrier dysfunction facilitates the interaction between systemic autoimmunity and CNS-resident immune mechanisms. Clinical studies provide evidence of barrier dysfunction, endothelial activation, CSF inflammation, neuroimaging abnormalities, and associations with selected autoantibodies. In contrast, animal and cell-based models provide mechanistic support for the roles of autoantibodies, immune complexes, complement activation, type I IFN signaling, leukocyte recruitment, astrocyte and microglial activation, and neuronal dysfunction. Nevertheless, the temporal sequence and relative contribution of these mechanisms in individual patients remain incompletely defined and require further validation in longitudinal human studies.

### PNS-NPSLE

Although CNS-NPSLE has been the primary focus of research in recent years, involvement of the peripheral nervous system (PNS) in SLE remains relatively underexplored [12]. Available clinical studies indicate that peripheral neuropathies occur in a subset of patients with SLE and may include polyneuropathy, mononeuropathy, cranial neuropathy, plexopathy, and small-fiber neuropathy [12, 101, 102]. However, compared with CNS-NPSLE, the immunopathological mechanisms underlying PNS involvement are less clearly defined, and most proposed pathways are inferred from clinical observations, studies of autoimmune neuropathies, skin biopsy findings, and limited histopathological reports.

The pathomechanisms underlying PNS-NPSLE may share similarities with those observed in other autoimmune neuropathies. In SLE, neuropathic processes are thought to reflect interactions between systemic autoimmunity, chronic inflammation, vascular injury, and local immune-mediated damage to peripheral nerves. Chronic inflammation can increase the production of inflammatory mediators, including IL-1β, IL-6, and TNF-α, which may sensitize nociceptive pathways and contribute to neuropathic pain [101]. In response to inflammatory or noxious stimuli, sensory neurons may release substance P, a neuropeptide involved in neurogenic inflammation. Substance P can promote vasodilation, increase vascular permeability, and stimulate immune cells, including macrophages and mast cells, to release additional proinflammatory mediators [103, 104]. These mechanisms may create a self-amplifying inflammatory feedback loop contributing to the persistence of neuropathic pain. However, the direct contribution of substance P to SLE-associated peripheral neuropathy remains incompletely established.

The blood–nerve barrier (BNB), which separates peripheral nerves from circulating immune cells and soluble inflammatory mediators, may represent an important interface between systemic autoimmunity and peripheral nerve injury. Experimental and pathological studies of autoimmune neuropathies suggest that the BNB is not uniformly impermeable and may be particularly vulnerable at distal nerve endings, nerve roots, dorsal root ganglia, and regions with specialized nodal or paranodal architecture [105, 106]. Increased BNB permeability may facilitate the entry of immune cells, autoantibodies, complement components, and cytokines into peripheral nerve compartments. By analogy with BBB disruption in CNS-NPSLE, BNB dysfunction may therefore contribute to PNS involvement in SLE, although direct evidence in SLE-associated neuropathy remains limited.

The nodes of Ranvier and paranodal regions are of particular interest because they contain highly organized molecular structures essential for saltatory conduction and may serve as antigenic targets in autoimmune neuropathies. Nodopathies are characterized by antibodies directed against nodal antigens, such as neurofascin-186 or pan-neurofascin, whereas paranodopathies involve antibodies against paranodal antigens, including neurofascin-155, contactin-1, contactin-associated protein 1, and pan-neurofascin [106]. These antibody-mediated neuropathies provide a useful conceptual framework for understanding immune-mediated peripheral nerve dysfunction. However, their specific relevance to PNS-NPSLE has not been firmly established, and currently available data are insufficient to define a SLE-specific nodal or paranodal antibody signature.

Immune complex deposition may represent another mechanism contributing to peripheral nerve injury in SLE. Histopathological evidence from case reports has demonstrated deposition of immune complexes within the endoneurium and associated vasculature, supporting a role for immune-mediated microvascular and endoneurial injury in selected patients [107]. This mechanism may, at least conceptually, resemble the autoimmune-inflammatory and vascular pathways described in CNS-NPSLE. However, immune complex deposition alone is unlikely to fully explain the heterogeneity of peripheral nerve involvement in SLE, which may also involve cytokine-mediated injury, autoantibody-associated dysfunction, vasculitis, complement activation, and ischemic or thrombotic vascular damage. Compared with CNS-NPSLE, direct evidence for thrombotic mechanisms in PNS-NPSLE is scarce; however, vascular injury, vasculitis, microvascular ischemia, immune complex deposition within endoneurial vessels, complement activation, and APLAs-related prothrombotic mechanisms may contribute to peripheral nerve damage in selected patients.

Multiple immune cell populations may participate in peripheral nerve inflammation. B lymphocytes, dendritic cells, macrophages, mast cells, and T lymphocytes can contribute to local inflammatory responses and progressive neural damage [101, 105]. T-cell-mediated orchestration of immune cell recruitment and activation may be particularly relevant, although direct evidence from SLE-associated peripheral nerve tissue remains limited. These processes may partially parallel inflammatory infiltration of vascular and barrier-associated structures observed in CNS-NPSLE, but the anatomical and immunological features of the PNS differ substantially from those of the CNS.

Additional support for immune-mediated peripheral nerve involvement comes from skin biopsy studies in patients with SLE. These studies have reported reduced intraepidermal nerve fiber density accompanied by inflammatory infiltrates, findings consistent with small-fiber neuropathy in selected patients [102]. Such observations suggest that distal sensory fibers may be particularly vulnerable to immune-mediated or inflammatory injury. They also indicate that mechanisms affecting distal small fibers may differ from those involved in proximal nerve roots, large myelinated fibers, or mixed sensorimotor neuropathies.

Depending on the types of fibers involved and the underlying pathological mechanisms, axonal polyneuropathy affecting sensory and motor fibers appears to be the most commonly reported neuropathy in patients with PNS involvement in SLE [12]. This observation suggests that PNS involvement in SLE is often generalized rather than restricted to a single functional fiber type. Nevertheless, the relative contribution of immune complex deposition, vasculitic injury, BNB dysfunction, autoantibody-mediated nodal or paranodal dysfunction, and cytokine-driven neuroinflammation remains insufficiently defined.

Overall, current evidence suggests that PNS-NPSLE may result from overlapping inflammatory, vascular, and antibody-mediated mechanisms. However, compared with CNS-NPSLE, the available evidence is more limited and largely associative. Further studies integrating clinical neurophysiology, skin biopsy, nerve imaging, CSF and serum biomarkers, and, when available, peripheral nerve histopathology are required to clarify the mechanisms of peripheral nervous system involvement in SLE. Together, CNS and PNS manifestations of NPSLE illustrate how systemic immune dysregulation in SLE may affect distinct anatomical compartments of the nervous system through partially overlapping but not identical inflammatory, vascular, barrier-related, and antibody-mediated mechanisms.

## Immune system involvement in NPSLE pathogenesis

Disturbances of innate and adaptive immunity are central to the pathogenesis of SLE, although the precise cellular and molecular mechanisms leading to neuropsychiatric involvement remain incompletely defined. Environmental and external factors, including UVB radiation, toxins, and infections, may exacerbate SLE activity by activating innate immune cells and promoting autoreactive immune responses [108, 109]. In susceptible individuals, this process contributes to the release and persistence of autoantigens, activation of autoreactive lymphocytes, production of autoantibodies, and formation of immune complexes. These events disrupt cellular and tissue homeostasis and may amplify inflammatory responses, ultimately contributing to organ damage [35, 42]. In NPSLE, this systemic autoimmune loop is thought to interact with vascular, endothelial, and CNS-resident immune mechanisms, although the temporal sequence of these events remains difficult to establish in humans.

Because evidence regarding immune system involvement in NPSLE is derived from heterogeneous sources, including clinical cohort studies, CSF biomarker analyses, histopathological reports, animal models, and in vitro or ex vivo experimental systems, the following sections distinguish between different levels of evidence. Accordingly, findings from human observational studies are framed as indicating associations rather than causality, whereas experimental data from animal and cell-based models are used to clarify selected mechanistic pathways. This distinction is particularly important because many mechanisms proposed in NPSLE, including complement-mediated BBB disruption, NETosis-related endothelial injury, IFN-I-driven neuroinflammation, lymphocyte recruitment to the choroid plexus, and microglial activation, are supported more strongly by experimental models than by direct causal evidence in patients.

Given the multifactorial nature of NPSLE, the following sections discuss the contribution of selected innate and adaptive immune components, including the complement system, neutrophils, plasmacytoid dendritic cells, T lymphocytes, and B lymphocytes. Where possible, we distinguish between findings derived from human observational studies and mechanisms supported by experimental models.

### The role of the complement system in the pathophysiology of NPSLE

The complement system plays an important role in humoral innate immunity and contributes to the regulation of adaptive immune responses. It comprises more than 30 soluble and membrane-associated proteins synthesized predominantly in the liver, although several immune and non-immune cell populations can also produce complement components locally [42, 44]. In SLE, the complement system has a dual role. On the one hand, complement is essential for the clearance of apoptotic material and immune complexes; on the other hand, excessive or dysregulated complement activation may amplify inflammation and tissue injury.

In the pathophysiology of SLE and NPSLE, one of the most relevant functions of complement is its involvement in immune complex handling. Defective clearance of apoptotic material and immune complexes may disrupt peripheral immune tolerance and promote autoimmunity. This concept is supported by clinical and genetic observations showing that individuals with homozygous or biallelic deficiencies in components of the classical complement pathway are at increased risk of developing SLE [43, 44]. In established SLE, circulating complement levels often correlate with immune complex formation, lymphocyte activation, disease activity, and tissue injury across multiple organs [44, 45, 47, 77, 110]. However, in clinical studies these associations do not necessarily prove that complement abnormalities directly cause neuropsychiatric manifestations.

In NPSLE, complement activation may be indirectly linked to neuropsychiatric manifestations through increased systemic disease activity, immune complex deposition, endothelial injury, and BBB dysfunction [9, 110]. Histopathological studies provide more direct evidence that complement activation may contribute to CNS injury in NPSLE. Brain tissue analyses from patients with SLE and NPSLE have demonstrated vasculopathy, microinfarcts, thrombi, and deposition of complement activation products, including C4d and C5b-9 [58, 59]. Case-based reports have additionally described immune complex vasculitis and platelet deposition in cerebral vessels [9, 60]. These observations support the involvement of complement-mediated vascular injury, although they are limited by the scarcity of CNS tissue samples and by the fact that available material often represents severe or fatal cases.

Particular attention has been given to the complement component C5a. Experimental lupus models and human in vitro BBB systems have shown that C5a–C5aR signaling can alter BBB integrity by affecting endothelial junctional proteins and increasing barrier permeability [81–83]. These studies provide mechanistic support for a causal role of C5a-mediated signaling in BBB disruption under experimental conditions. In humans, however, the evidence is more indirect and derives mainly from serum complement measurements, CSF or peripheral biomarker studies, and histopathological observations. Elevated serum C5a levels have been reported in patients with NPSLE [111], but such findings should be interpreted as associative rather than definitive evidence that C5a directly causes neuropsychiatric manifestations.

Complement activation may also contribute to thrombotic processes, particularly in patients with concomitant APS. Enhanced deposition of complement activation products on platelets has been associated with thrombotic events in SLE, especially in the presence of antiphospholipid antibodies and anti-β2-glycoprotein I antibodies [112]. This observation is consistent with the thromboinflammatory mechanisms implicated in focal NPSLE, although the relative contribution of complement activation, aPL-mediated platelet activation, endothelial dysfunction, and traditional vascular risk factors may vary among patients.

Although experimental studies support the pathogenic relevance of the C5a–C5aR axis in BBB dysfunction and inflammatory cell recruitment [81–84], direct therapeutic evidence targeting this pathway in human NPSLE remains lacking. Therefore, complement inhibition represents a biologically plausible therapeutic approach, but its clinical relevance to the neuropsychiatric manifestations of SLE requires dedicated translational and clinical studies.

Increasing evidence also indicates a bidirectional interaction between the complement system and LDGs [36]. Neutrophils express receptors for complement components, including CR1, C3aR, and C5aR1, which enable their activation by complement-derived signaling molecules [78]. This activation may enhance phagocytic activity, chemotaxis, and NETosis. Conversely, NETosis-derived products can stimulate complement activation, thereby maintaining a self-amplifying inflammatory loop [113].

In NPSLE, C5a may be particularly relevant because it can promote neutrophil chemotaxis and facilitate inflammatory cell recruitment toward CNS-associated vascular and barrier structures [84, 114]. Mechanistically, C5a–C5aR1 signaling can stimulate endothelial cells to express adhesion molecules such as ICAM-1 and VCAM-1, thereby promoting adhesion and transmigration of neutrophils, monocytes, and lymphocytes [78, 81–84]. This process may contribute to BBB dysfunction and support the autoimmune-inflammatory pathway of NPSLE.

Within the CNS, complement activation may also influence resident immune cells. Experimental studies suggest that complement-derived signals can activate microglia, astrocytes, and possibly neurons through proinflammatory pathways, including NF-κB-related signaling [83]. However, direct evidence defining the role of complement-mediated glial activation in human NPSLE remains limited. Therefore, complement should be regarded as a biologically plausible and experimentally supported contributor to NPSLE pathogenesis, with the strongest human evidence coming from histopathological, biomarker, and vascular association studies.

### The role of neutrophils and plasmacytoid dendritic cells in the pathophysiology of NPSLE

Neutrophils are the most abundant leukocytes in human blood and constitute an essential component of the innate immune response. Despite their short lifespan, neutrophil homeostasis is maintained through continuous production and release from the bone marrow. Their participation in inflammatory responses involves adhesion to activated vascular endothelium, transendothelial migration, chemotaxis toward inflammatory sites, phagocytosis, production of reactive oxygen species, degranulation, and formation of neutrophil extracellular traps [37, 38, 115].

A distinct neutrophil population, commonly referred to as LDGs, has been identified in patients with SLE [36, 116]. These cells display an increased propensity for spontaneous NETosis and production of inflammatory mediators, including type I IFNs and TNF-α [36, 41]. In SLE, NETosis-derived nuclear and mitochondrial material is highly immunogenic and may serve as a source of danger-associated molecular patterns and autoantigens [35–39, 41]. Although these mechanisms are well supported in systemic SLE, their direct contribution to CNS injury in human NPSLE remains less clearly established and is largely inferred from translational studies, experimental models, and the known effects of NET-derived mediators on endothelial and vascular integrity.

Mechanistic studies indicate that mitochondrial DNA released within NETs may be particularly immunostimulatory because it contains hypomethylated CpG motifs resembling bacterial DNA. Oxidized mitochondrial DNA released within NETs can activate DNA-sensing pathways, including STING, whereas mitochondrial nucleoids derived from SLE neutrophils may stimulate TLR9 signaling and promote type I IFN responses [36]. These findings provide mechanistic support for the link between neutrophil dysregulation, nucleic acid sensing, and IFN-I activation in SLE. However, the extent to which these pathways operate directly within the CNS of patients with NPSLE remains uncertain.

LDGs may also contribute to vascular and barrier dysfunction by releasing matrix metalloproteinases, including MMP-9, which can degrade components of the vascular basement membrane and compromise vascular integrity [67, 117]. Experimental ischemia models and human post-mortem analyses of ischemic brain injury have shown that MMP-9-positive neutrophil infiltration is associated with BBB damage [68, 118]. Although these studies are not specific to NPSLE, they provide mechanistic support for a pathway through which neutrophil-derived mediators may contribute to BBB disruption in inflammatory or thrombo-ischemic CNS injury.

NET-associated proteases, including neutrophil elastase and cathepsin G, can process and activate inflammatory cytokines such as IL-1α and IL-36, thereby amplifying inflammatory responses [119]. In vitro and translational studies further indicate that NETs can induce endothelial activation, tissue factor expression, and endothelial dysfunction, processes relevant to both vascular inflammation and thrombosis [120–122]. Therefore, neutrophil dysregulation may link the autoimmune-inflammatory and thrombo-ischemic pathways of NPSLE.

NETosis-derived material can activate plasmacytoid dendritic cells through immune complexes containing nucleic acids. These complexes may be internalized through Fc receptor-dependent mechanisms and recognized by endosomal TLR7 and TLR9, leading predominantly to type I IFN production [48, 78]. Type I IFNs subsequently promote activation of neutrophils, dendritic cells, B cells, and T cells, thereby maintaining the self-amplifying autoimmune loop characteristic of SLE [39, 41, 42, 48]. In the context of NPSLE, this pathway provides a plausible mechanistic link between systemic autoimmunity, endothelial activation, BBB dysfunction, and CNS inflammation, although direct causal evidence in patients remains limited.

Type I IFN signaling may also contribute to endothelial dysfunction. Experimental studies indicate that IFN-α can upregulate IFN-stimulated genes in neutrophils and enhance their capacity to damage vascular endothelium [41, 123]. Cytokines released by dendritic cells, including IFN-α, TNF-α, and IL-6, may stimulate BBB endothelial cells, increase adhesion molecule expression, and promote vascular permeability. These processes provide a plausible mechanistic link between systemic IFN-I activation, immune cell migration into the CNS, and neuroinflammatory changes in NPSLE [48, 86, 123–125].

Consequently, neutrophils and plasmacytoid dendritic cells may cooperate to establish an inflammatory network that promotes oxidative stress, endothelial activation, BBB dysfunction, and CNS immune activation. In experimental systems, inflammatory mediators such as IFN-I, IL-1, IL-6, and TNF-α may influence microglia and astrocytes, contributing to neurotoxicity and neuronal dysfunction [86, 94–96, 100, 124, 125]. However, in human NPSLE, the direct contribution of neutrophil–pDC interactions to CNS pathology remains incompletely defined and requires further validation.

Therapeutic blockade of type I IFN signaling with anifrolumab, a monoclonal antibody targeting the type I IFN receptor, has demonstrated efficacy in systemic SLE and may reduce IFN-driven inflammatory pathways. Based on its mechanism of action, anifrolumab could theoretically attenuate pDC-driven IFN-I signaling, neutrophil activation, NETosis, and downstream cytokine production [126]. Preliminary clinical observations and case-based analyses suggest that IFN-I pathway inhibition may be relevant to selected neuropsychiatric manifestations, but dedicated clinical studies in NPSLE are still lacking [127]. Therefore, its role in NPSLE should currently be considered promising but unproven.

Importantly, β2-glycoprotein I has been identified on the surface of neutrophils, and IgG anti-β2-glycoprotein I antibodies can induce NETosis in human neutrophils [128]. This provides a mechanistic basis for the involvement of antiphospholipid antibodies in neutrophil-driven inflammation and thrombosis. NETosis may therefore contribute both to tissue injury and thrombus formation [129, 130], supporting a possible role for LDGs in the thrombo-ischemic pathway of NPSLE. Nevertheless, the extent to which this mechanism directly explains neuropsychiatric manifestations in individual patients remains to be determined.

### The role of T and B lymphocytes in the pathophysiology of NPSLE

Innate immune mechanisms are not the only processes involved in the immunopathogenesis of SLE and NPSLE. Adaptive immune cells, particularly T and B lymphocytes, are central to the loss of tolerance, autoantibody production, immune complex formation, and persistence of chronic inflammation. In SLE, T lymphocytes provide help to autoreactive B cells, thereby promoting antinuclear antibody production [131]. Numerous phenotypic and functional abnormalities of T cells have been described in patients with SLE, including altered signaling, increased activation, abnormal cytokine production, and impaired regulatory function [132].

Although studies specifically investigating T-cell subsets in NPSLE remain limited, extensive evidence from SLE cohorts indicates abnormalities in T-cell activation, signaling, and regulatory function [131, 132]. Regulatory T cells are essential for maintaining immune homeostasis by suppressing autoreactive T and B cells and limiting excessive inflammatory responses [133]. Meta-analytic and clinical studies have reported reduced proportions or impaired function of Tregs in patients with SLE [134]. These findings support an association between T-cell dysregulation and SLE activity, but direct evidence linking Treg deficiency to human NPSLE is currently insufficient.

The imbalance between Th1, Th2, Th17, and Treg populations may have important pathogenic consequences. In healthy individuals, Tregs suppress excessive production of proinflammatory cytokines, including IFN-γ and IL-17, and help maintain equilibrium between helper T-cell subsets. In SLE, this balance is disrupted, which promotes inflammatory cytokine production, B-cell activation, and autoantibody generation [51, 52]. Th17 differentiation is promoted by cytokines such as IL-6, TGF-β, IL-1β, and IL-23, which may be produced by dendritic cells and other innate immune cells [52]. The transcription factor RORγt is a key regulator of Th17 differentiation and controls the expression of signature cytokines, including IL-17 [53]. IL-17 can activate endothelial cells and fibroblasts, induce production of IL-6, IL-8, and TNF-α, enhance adhesion molecule expression, promote neutrophil recruitment, and stimulate B-cell activation and maturation [50–53]. These mechanisms may contribute to persistent inflammation and autoantibody production in SLE.

Experimental studies provide mechanistic support for the role of Th17-related cytokines in BBB dysfunction and CNS inflammation. IL-17 and IL-22 can increase BBB permeability by altering endothelial cell function and junctional proteins [54–56]. Studies using experimental CNS inflammation models have shown that IL-17A promotes migration of lymphocytes and neutrophils into the CNS and exacerbates neuroinflammatory injury [135]. In vitro studies further suggest that IL-17 can activate astrocytes and induce production of inflammatory mediators such as IL-6 and TNF-α [136]. These findings support a biologically plausible role for Th17-related pathways in NPSLE, although direct human evidence remains inconclusive.

B lymphocytes play a multifaceted role in SLE and may contribute to NPSLE through several mechanisms. First, autoreactive B cells produce antinuclear and other autoantibodies that can form immune complexes and promote tissue inflammation. Second, B cells function as antigen-presenting cells that activate T lymphocytes and amplify adaptive immune responses. Third, B-cell-derived autoantibodies may contribute to neuronal or endothelial dysfunction, particularly under conditions of BBB disruption. However, in human NPSLE, the degree to which B cells infiltrate the CNS and participate in local immune responses remains incompletely defined.

Indirect evidence supports local humoral immune activation in CNS-NPSLE. Intrathecal immunoglobulin synthesis, reflected by an elevated IgG index and the presence of oligoclonal bands in CSF, has been reported in patients with SLE and neuropsychiatric manifestations [33, 137]. Comparative analyses suggest that additional IgG bands are more frequently detected in individuals with CNS involvement than in those without neuropsychiatric manifestations [137]. These observations support local or compartmentalized immune activation, but they do not by themselves define the precise cellular source of intrathecal immunoglobulin production.

Autoantibodies produced by B cells may serve as potential biomarkers of NPSLE, including anti-NMDAR, anti-ribosomal P, anti-ganglioside, anti-endothelial, antiphospholipid, and other neuronal or glial antigen-directed antibodies [17–27, 70, 71]. However, their clinical utility remains limited because sensitivity and specificity vary across patient populations, neuropsychiatric phenotypes, biological compartments, and study designs. Therefore, although B-cell-derived autoantibodies are strongly implicated in NPSLE, their diagnostic and pathogenic significance should be interpreted in relation to BBB integrity, CSF findings, clinical phenotype, and supporting experimental data.

The CXCR4–CXCL12 axis may also contribute to lymphocyte trafficking in SLE. Dysregulated CXCR4/CXCL12 expression has been reported in subsets of patients with SLE and has been associated with disease activity [138]. Although this pathway may be relevant to immune cell recruitment to inflamed tissues, its specific role in lymphocyte trafficking to CNS-associated barriers in human NPSLE remains to be directly demonstrated.

Human histopathological studies are limited because CNS tissue samples from patients with NPSLE are rarely available. Nevertheless, available reports describe vasculopathic alterations, microinfarctions, small-vessel injury, and perivascular lymphocytic infiltrates [58–60]. These findings support the involvement of vascular and inflammatory mechanisms, but they do not fully define the relative contribution of B cells, T cells, or other immune populations.

Experimental studies suggest that chronic inflammation in NPSLE may promote leukocyte recruitment to the choroid plexus and support the formation of tertiary lymphoid-like structures within CNS-associated barrier compartments. Murine studies have shown that choroid plexus-infiltrating CD4-positive T cells can contribute to NPSLE-like manifestations through local inflammatory signaling and microglial activation by IFN [87, 88]. In experimental models, depletion of CD4-positive T cells or interference with adhesion molecule-dependent recruitment can reduce lymphoid infiltrates and improve neuropsychiatric-like symptoms [87, 88]. These findings provide important mechanistic insight, but their translation to human NPSLE remains to be established.

## Integrated immunopathogenesis of NPSLE

**Fig. 2:**
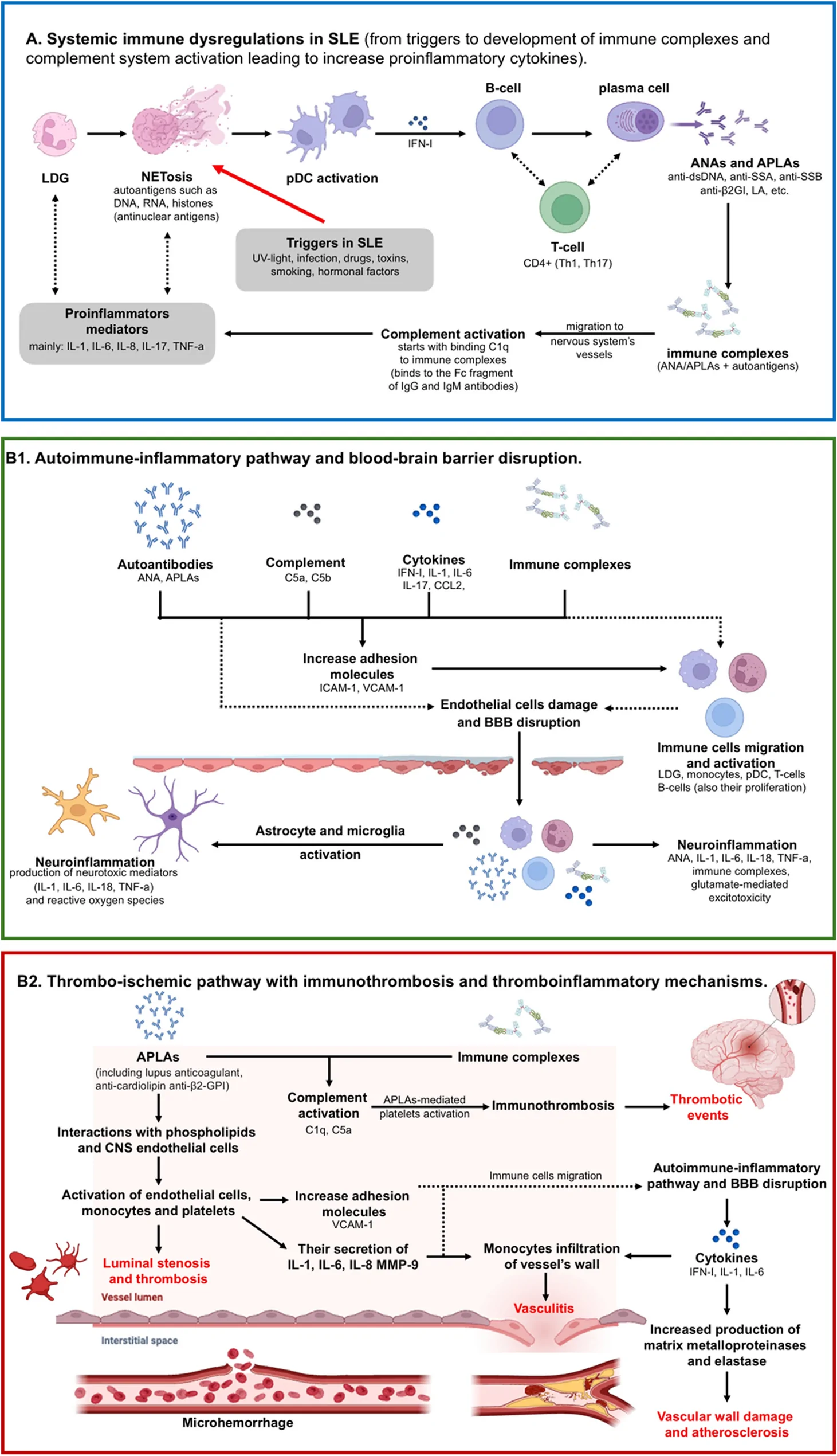
Integrated immunopathogenesis of neuropsychiatric systemic lupus erythematosus (NPSLE). **A** Systemic immune dysregulation in systemic lupus erythematosus (SLE). Environmental and endogenous triggers, including UV exposure, infections, drugs, toxins, smoking, and hormonal factors, promote neutrophil activation and NETosis, resulting in the release of autoantigens such as DNA, RNA, and histones. These autoantigens activate plasmacytoid dendritic cells (pDCs), leading to type I interferon (IFN-I) production and amplification of autoreactive B- and T-cell responses. Activated B cells differentiate into plasma cells producing autoantibodies, including antinuclear antibodies (ANAs) and antiphospholipid antibodies (APLAs). Formation of immune complexes and complement activation contributes to systemic inflammation characterized by increased levels of proinflammatory cytokines. **B1** Autoimmune-inflammatory pathway and blood–brain barrier (BBB) disruption in NPSLE. Autoantibodies, complement components, cytokines, and immune complexes promote endothelial activation, increased expression of adhesion molecules, and BBB dysfunction. BBB disruption facilitates migration of peripheral immune cells, including low-density granulocytes (LDGs), activated T cells, B cells, monocytes, and pDCs, into the central nervous system (CNS). Local activation of astrocytes and microglia leads to production of neurotoxic mediators, reactive oxygen species, and additional proinflammatory cytokines, thereby amplifying neuroinflammation and neuronal dysfunction. **B2** Thrombo-ischemic pathway with immunothrombosis and thrombo-inflammatory mechanisms in NPSLE. Antiphospholipid antibodies and immune complexes interact with phospholipids and endothelial cells, promoting activation of endothelial cells, platelets, and monocytes. Complement activation further enhances platelet activation and immunothrombosis. These processes contribute to luminal stenosis, thrombosis, vasculitis, microhemorrhages, vascular wall injury, and atherosclerotic changes. Increased production of cytokines, matrix metalloproteinases, and elastases further destabilizes vascular integrity and promotes BBB disruption and immune cell infiltration, linking thrombo-inflammatory and autoimmune-inflammatory mechanisms in NPSLE

The pathogenesis of NPSLE is multifactorial and involves complex interactions among systemic immune dysregulation, vascular injury, BBB dysfunction, and local neuroinflammatory processes. Although individual mechanisms are often discussed separately, current evidence suggests that they operate as interconnected elements of a pathogenic network rather than isolated pathways (Fig. 2).

Importantly, thrombo-ischemic and autoimmune-inflammatory mechanisms frequently coexist and act synergistically. This interaction contributes to the heterogeneity of clinical manifestations observed in patients with NPSLE. Focal manifestations are more strongly linked to vascular injury, thrombosis, and antiphospholipid antibodies, whereas diffuse manifestations are more often associated with inflammatory mediators, BBB dysfunction, autoantibodies, and neuroimmune activation. Nevertheless, these categories overlap substantially, particularly at the level of the neurovascular unit and CNS barriers.

Understanding this integrated pathogenic network is essential for identifying reliable biomarkers and developing targeted therapeutic strategies for NPSLE. Future approaches should therefore combine clinical phenotyping, attribution models, neuroimaging, CSF analysis, autoantibody profiling, and molecular immune signatures.

### Limitations of the current knowledge

Despite substantial progress in understanding the immunopathogenesis of NPSLE, several important limitations remain. First, the marked clinical heterogeneity represents a major challenge in both research and clinical practice. Neuropsychiatric manifestations range from mild cognitive dysfunction and mood disorders to severe neurological syndromes such as seizures, cerebrovascular disease, psychosis, and acute confusional states. Distinguishing primary immune-mediated NPSLE from secondary neurological complications, including infection, metabolic disturbances, treatment-related toxicity, and traditional vascular disease, remains difficult. Differences in diagnostic criteria, attribution models, study populations, and definitions of neuropsychiatric events contribute to the wide variability in reported prevalence and clinical characteristics.

Second, many mechanistic insights into NPSLE pathogenesis originate from animal models or cell-based experimental systems. These models provide valuable information on BBB disruption, complement activation, microglial responses, autoantibody-mediated neuronal injury, IFN-I signaling, and choroid plexus inflammation. However, they reproduce only selected aspects of the human disease and cannot fully reflect the clinical, immunological, vascular, and neuropsychiatric complexity of NPSLE. Therefore, mechanisms demonstrated in experimental models should be interpreted as biologically plausible and mechanistically informative, but not automatically equivalent to proven causal pathways in humans.

Third, direct investigation of CNS tissue in patients with NPSLE is extremely limited. Brain biopsies are rarely performed because of the invasiveness of the procedure and ethical considerations, and post-mortem studies usually represent severe or fatal cases. Consequently, most human studies rely on indirect measures, including neuroimaging, CSF analysis, serum biomarkers, and peripheral immune profiling. Although these approaches provide important information, they often cannot establish the temporal sequence of events or distinguish primary CNS immune activation from secondary consequences of systemic inflammation, vascular injury, or treatment effects.

Fourth, the role of microglia in human NPSLE remains insufficiently defined. Animal models suggest that microglial activation may contribute to synaptic dysfunction, neuronal injury, behavioral abnormalities, and cognitive impairment. However, direct assessment of microglial activation in patients is challenging. CSF studies and neuroimaging may provide indirect evidence of CNS inflammation, but they cannot fully define the origin, phenotype, and functional state of microglia in affected brain regions. Further studies integrating advanced neuroimaging, CSF proteomics, transcriptomics, and carefully selected clinical phenotypes are needed to clarify the role of microglia in human disease.

Fifth, many proposed biomarkers of NPSLE, including autoantibodies, cytokines, complement components, endothelial activation markers, and CSF inflammatory mediators, show limited diagnostic specificity and sensitivity. Their performance varies across patient populations, neuropsychiatric manifestations, sample types, disease activity states, and attribution models. This lack of reliable biomarkers hampers early diagnosis, complicates assessment of disease activity, and limits evaluation of treatment response.

An additional limitation of the current literature is the heterogeneity of evidence supporting individual pathogenic mechanisms. Some mechanisms, such as the association between antiphospholipid antibodies and thrombotic or ischemic events, are supported by relatively strong clinical and epidemiological evidence. Others, including microglial activation, complement-mediated BBB disruption, choroid plexus lymphocyte infiltration, and NETosis-driven endothelial injury, are supported mainly by animal models, in vitro studies, or indirect translational evidence. Although these experimental systems provide important mechanistic insight, they do not fully reproduce the complexity of human NPSLE. Therefore, proposed pathways should be interpreted according to the type and strength of supporting evidence, and causal conclusions in humans should be made cautiously.

Addressing these limitations will require well-designed multicenter studies, standardized attribution models for neuropsychiatric symptoms, and integration of clinical, imaging, and molecular data.

## Future perspectives in NPSLE research

Research on NPSLE is evolving rapidly, and several directions may improve understanding, diagnosis, and treatment of this complex condition. A major priority is the identification of reliable biomarkers that can support early diagnosis, phenotype stratification, disease monitoring, and evaluation of treatment response. Because individual biomarkers are unlikely to capture the full complexity of NPSLE, future studies should focus on integrated biomarker panels combining autoantibody profiles, complement markers, cytokines, endothelial activation markers, CSF parameters, neuroimaging data, and molecular immune signatures.

Advances in transcriptomics, proteomics, metabolomics, and single-cell technologies may help identify immune signatures associated with specific NPSLE phenotypes. Such approaches should ideally be applied to well-characterized patient cohorts using standardized attribution models and longitudinal sampling. Integration of peripheral blood, CSF, and neuroimaging data may allow distinction between predominantly inflammatory, thrombotic, vascular, and mixed pathogenic patterns.

Another promising research direction involves the neurovascular unit and CNS barrier systems. Better understanding of how systemic inflammation, complement activation, NETosis, antiphospholipid antibodies, and endothelial dysfunction alter BBB and blood–CSF barrier integrity may reveal therapeutic targets aimed at protecting the CNS from immune-mediated injury. Targeted immunotherapies also represent an important area of future investigation. Biological agents modulating type I IFN signaling, complement activation, B-cell function, T-cell costimulation, or cytokine networks may have therapeutic potential in selected patients with NPSLE. Although some of these approaches have demonstrated efficacy in systemic SLE, their specific impact on neuropsychiatric manifestations remains insufficiently studied. Dedicated clinical trials or carefully designed observational studies are needed to determine whether these therapies improve CNS-specific outcomes.

Growing attention is also being paid to microglia and neuroinflammation. Therapeutic strategies aimed at modulating microglial activation, reducing synaptic injury, or limiting neurotoxic inflammatory responses may represent innovative approaches to preventing neuronal dysfunction. However, because most current evidence derives from animal models, human validation will be essential before these strategies can be translated into clinical practice.

Future studies integrating rheumatology, neurology, immunology, neuroimaging, and molecular profiling will be crucial for developing a comprehensive understanding of NPSLE pathogenesis and translating mechanistic discoveries into effective therapeutic strategies.

## Conclusions

NPSLE represents one of the most complex and severe manifestations of SLE. Its pathogenesis involves an intricate interplay between systemic autoimmunity, vascular injury, BBB dysfunction, and neuroinflammatory processes. Current evidence implicates autoantibodies, immune complex deposition, complement activation, NETosis, type I IFN signaling, endothelial dysfunction, lymphocyte dysregulation, and microglial activation in the development of neuropsychiatric manifestations.

However, the strength of evidence differs across these mechanisms. Human clinical studies provide important associations between immune abnormalities and NPSLE phenotypes, whereas histopathological studies offer direct but limited evidence of CNS vascular and inflammatory injury. Animal models and in vitro systems provide mechanistic support for selected pathways, but they do not fully reproduce the complexity of human NPSLE. Therefore, many proposed mechanisms should be viewed as biologically plausible and experimentally supported, but not yet definitively established as causal in patients.

The coexistence of autoimmune-inflammatory and thrombo-ischemic pathways contributes to the heterogeneity of clinical manifestations observed in NPSLE. This complexity, together with the lack of reliable biomarkers and standardized attribution models, continues to challenge diagnosis and management [139]. Further research integrating clinical phenotyping, neuroimaging, CSF analysis, and molecular immune profiling is required to refine diagnosis, identify actionable biomarkers, and develop targeted therapeutic strategies for patients with NPSLE.

## Data Availability

No datasets were generated or analysed during the current study.
